# A One-Year Longitudinal Study Examining the Direct and Indirect Effects of AI Dependence on Work Engagement and Gender Differences

**DOI:** 10.3390/bs16010044

**Published:** 2025-12-24

**Authors:** Jiani Wen, Yuju Lei, Qingqi Liu

**Affiliations:** 1 Faculty of Psychology, Beijing Normal University, Beijing 100875, China; fionawenjiani@outlook.com (J.W.); liuqingqi@bnu.edu.cn (Q.L.); 2 School of Teacher Education, Hubei University of Arts and Science, Xiangyang 441053, China; 3 Department of Psychology, Beijing Normal University at Zhuhai, Zhuhai 519087, China

**Keywords:** AI dependence, work self-efficacy, work engagement, gender differences, longitudinal study

## Abstract

As artificial intelligence (AI) products become increasingly integrated into daily life, AI dependence has gained significant public and scholarly attention. While existing research has primarily examined its impact on students, few studies have investigated its association with employee career development, particularly work engagement. Our one-year longitudinal study involving 1108 employees aged 21–60 examined the long-term effect of AI dependence on work engagement, incorporating work self-efficacy as a mediator and gender as a moderator. Using three-wave survey data, we found that AI dependence at Time 1 (T1) directly predicted work engagement at Time 3 (T3), and also exerted a significant indirect effect through work self-efficacy at Time 2 (T2). While the mediating effect of T2 work self-efficacy showed no gender differences, the direct effect of T1 AI dependence on T3 work engagement was significantly stronger among male employees. These findings systematically address questions regarding the long-term, mediated, and gender-differentiated effects of AI dependence. They provide important warnings regarding AI dependence prevention and deliver practical implications for maintaining and enhancing employee self-efficacy and engagement in the AI era.

## 1. Introduction

The widespread adoption of artificial intelligence (AI) has given rise to a phenomenon termed “AI dependence,” which is increasingly observable across various sectors (Arora, 2025; Naseer et al., 2025; S. Huang et al., 2024; Yankouskaya et al., 2025). AI dependence denotes individuals’ irrational reliance on artificial intelligence tools in technological applications, resulting in diminished autonomy, a redistribution of decision-making authority, and heightened exposure to potential risks (Hu et al., 2023; S. Huang et al., 2024; Laestadius et al., 2024). The implications of AI dependence are particularly pronounced in contexts of rapid technological advancement, such as among students who use AI for assignment generation (Morales-García et al., 2024) and employees who rely on algorithmic decisions in their work (Zhao et al., 2025). AI dependence is associated with a range of negative consequences. Overreliance on AI in academic settings can negatively impact students’ development of essential competencies such as communication skills, collaboration abilities, problem-solving, and self-regulation (Zhai et al., 2024; D. Zhang et al., 2025). The tendency of AI to provide simplified responses may also inhibit the cultivation of critical and creative thinking skills among college students (Lin & Chen, 2024). Furthermore, dependence on AI may exacerbate procrastination (Mukhtar et al., 2025) and significantly affect students’ mental health, particularly regarding depression (X. Zhang et al., 2025).

While preliminary research has explored the impact of AI dependence on student populations, empirical investigations into its effects on employees are markedly limited. A pertinent question arises as to whether reliance on AI significantly predicts lower levels of work engagement among employees. Although there are some subjective opinions suggesting that AI dependence may weaken work engagement, rigorous empirical research comprehensively exploring the complex relationship between AI dependence and work engagement is still extremely lacking. Key unanswered questions include whether a longitudinal association exists, how the two variables are correlated, and whether their relationship varies by gender. The present study aims to investigate the longitudinal relationship between AI dependence and employee work engagement, while also examining the mediating role of work self-efficacy and the moderating role of gender.

### 1.1. AI Dependence and Work Engagement

Work engagement is defined as the sustained psychological investment that employees make in their work, characterized by high energy, strong concentration, and a profound sense of job identity (Bakker & Demerouti, 2008; Schaufeli & Bakker, 2010). Researchers posit that work engagement encompasses three dimensions: first, vigor, which refers to the abundant energy and psychological resilience displayed in the workplace, enabling individuals to operate with high efficiency in the face of challenges; second, dedication, which signifies the recognition of the meaningfulness of work and emotional commitment, motivating individuals to pursue goals and experience a sense of achievement; and third, absorption, characterized by a deep immersion in tasks, during which the perception of time diminishes and external distractions are reduced (Schaufeli & Bakker, 2010; Schaufeli et al., 2006). The three-dimensional construct of work engagement has also been widely validated across different populations in various countries (e.g., Balducci et al., 2010; Fong & Ng, 2012; Mills et al., 2012; Shimazu et al., 2008). Work engagement is highly susceptible to technological use, particularly regarding technology dependency. For example, a series of studies has demonstrated that dependence on social media has a considerable negative effect on work engagement (e.g., Boukari et al., 2025; J. Huang et al., 2024; Ibrahim et al., 2022; Khan et al., 2022).

While the integration of AI offers significant conveniences to employees, its powerful capabilities may lead to an increased reliance on the technology. Should employees become overly dependent on AI, all three dimensions of their work engagement may be adversely impacted. First, the use of AI tools to perform various work skill operations, such as automated report generation, may lead employees to enter a “low-energy” state of idleness due to the absence of challenging tasks, ultimately resulting in a gradual diminishment of their vitality. Second, when employees depend on AI to generate core solutions or assume a leading role in decision-making processes, they may perceive themselves as reduced to mere “tool operators.” As a result, their sense of purpose within their roles may diminish, negatively impacting their recognition of the value of their work and their willingness to contribute. Third, overreliance on AI for direct problem-solving without engaging in critical analysis can lead to fragmented attention and a decline in deep thinking capabilities (George et al., 2024; H. P. Lee et al., 2025), making it increasingly challenging to concentrate on subsequent tasks. It is important to note that the inferences made above are based on general observations, and the relationship between AI dependence and work engagement has not been directly validated through empirical research. Therefore, additional empirical studies are necessary to provide direct support for these claims.

### 1.2. Mediating Role of Work Self-Efficacy

Self-efficacy refers to an individual’s confidence in their ability to complete specific tasks or achieve particular goals (Bandura, 2006). Work self-efficacy specifically denotes the application of general self-efficacy within the workplace context (Raelin et al., 2011). According to goal-setting theory (Locke & Latham, 2006, 2019), individuals with high self-efficacy are more likely to establish challenging goals and commit to sustained efforts to attain them, thereby fostering high levels of work engagement. Additionally, from the perspective of conservation of resources theory (Hobfoll et al., 2018), self-efficacy serves as a psychological resource that equips individuals to effectively navigate work-related stress, which mitigates resource depletion and contributes to the maintenance of work engagement (Lloyd et al., 2017; Rafiei et al., 2024; Pei et al., 2024). A substantial body of research indicates that employees with higher self-efficacy demonstrate greater confidence in their job tasks, exhibit increased engagement in their work (Chaudhary et al., 2012; Orgambídez et al., 2020; Song et al., 2018), and generally attain higher job performance while experiencing greater job satisfaction (Caesens & Stinglhamber, 2014; Tian et al., 2019).

Excessive reliance on artificial intelligence (AI) products may adversely affect employees’ work self-efficacy. Drawing from social cognitive theory (Bandura, 2001), self-efficacy develops through mastery experiences. When AI undertakes critical tasks, it diminishes individuals’ opportunities for successful experiences, thereby undermining their self-efficacy. Additionally, prolonged dependence on AI may lead individuals to engage less in independent thinking and skill development, resulting in a decline in essential competencies such as problem-solving and decision-making abilities (Naseer et al., 2025; D. Zhang et al., 2025). Furthermore, according to social comparison theory (Caliskan et al., 2024; Festinger, 1954), employees may evaluate their skills in relation to those of AI, potentially inducing feelings of inferiority or what is termed “displacement threat,” which ultimately erodes their self-efficacy. A recent study indicated that exposure to AI-generated content correlates positively with social comparison, which, in turn, was negatively associated with both self-esteem and body image satisfaction (Tufail et al., 2024). Considering that AI dependence is a significant predictor of employees’ work efficacy and that work efficacy is significantly associated with work engagement, it can be inferred that work self-efficacy acts as a vital mediating variable between AI dependence and work engagement.

### 1.3. Gender Differences in AI Dependence and Work Engagement

While AI dependence theoretically has the potential to significantly predict work involvement, its effects are likely not static. Specific factors may influence the relationship between AI dependence and work engagement, resulting in variations in both strength and direction. Notably, gender may be influential, indicating that the relationship between AI dependence and work engagement may differ across different gender groups. Regarding the role of gender, while there is currently no strong empirical evidence supporting a gender difference in AI dependence, research on work engagement has yielded relatively consistent findings regarding gender differences. Work engagement is a gendered construct, with research indicating that men often exhibit higher levels of work engagement than women (Banihani et al., 2013; Steyn & Grobler, 2016; Rožman et al., 2021). The comparatively lower levels of work engagement among women may be attributed to their increased vulnerability to negative environments, such as those in family and workplace settings, which can hinder their engagement relative to their male counterparts (Lu & Wang, 2023; Metin Camgoz et al., 2016; Tian et al., 2021). As a potential factor that may be significantly associated with career development, AI dependence may have a stronger association with work engagement in women than in men. However, an alternative scenario may also exist; the impact of AI dependence on women might be diminished due to the influence of other negative factors they encounter. Despite the potential moderating role of gender on the direct or indirect effects of AI dependence on employee work engagement, current evidence remains insufficient to ascertain whether these predictive effects and their indirect pathways are stronger for males or females.

### 1.4. The Present Study

With the growing prevalence of AI tools, AI dependence has emerged as a phenomenon attracting considerable attention across various sectors of society. Although anecdotal evidence suggests that AI dependence may impact students, there is a notable dearth of scientifically rigorous quantitative research examining its effects on employee work engagement. Moreover, previous studies have largely utilized cross-sectional questionnaire designs, which are insufficient for thoroughly evaluating the predictive effects of AI dependence, especially regarding long-term outcomes and the mechanisms that underpin these relationships. The present study seeks to investigate the longitudinal effects of AI dependence on work engagement, with a specific focus on the mediating role of work self-efficacy and the potential gender differences in both the direct and indirect pathways from AI dependence to work engagement. Building on established theoretical frameworks and empirical findings, this study proposes four research hypotheses:

**Hypothesis** **1.** *AI dependence will exert a longitudinal effect on employee work engagement.*

**Hypothesis** **2.** *Work self-efficacy will mediate the longitudinal relationship between AI dependence and employee work engagement.*

**Hypothesis** **3.** *Gender will moderate the longitudinal relationship between AI dependence and employee work engagement.*

**Hypothesis** **4.** *Gender will moderate the mediating effect of work self-efficacy in the longitudinal relationship between AI dependence and employee work engagement.*

## 2. Materials and Methods

### 2.1. Participants

The present study was approved by the Ethics Committee of the first author’s university. Informed consent was obtained from all participants. Data were collected through three surveys conducted over the course of one year. Participants were recruited through the human resources departments of six companies, which distributed the recruitment notice across their respective subsidiaries. We invited full-time employees with experience in using AI products to participate in the study. Initially, 1212 employees from internet companies, pharmaceutical firms, publishing groups, and high schools participated in the first survey conducted at Time 1 (T1, June 2024). Six months later, at Time 2 (T2, December 2024), 1156 employees completed the second survey. Following another six-month interval, at Time 3 (T3, June 2025), 1108 employees participated in the third survey. Participant attrition during the second and third survey waves was primarily due to employee turnover and an unwillingness to continue. Comparisons between participants who completed all three waves and those who dropped out after T1 revealed no significant differences in T1 AI dependence (*t* = 0.65, *p* = 0.52) or T1 work engagement (*t* = 0.41, *p* = 0.68). Similarly, comparisons with those who dropped out after T2 showed no significant differences in T1 AI dependence (*t* = −1.71, *p* = 0.09), T1 work engagement (*t* = 0.92, *p* = 0.36), T2 work self-efficacy (*t* = 1.50, *p* = 0.14), or T3 work engagement (*t* = 1.32, *p* = 0.19). Among the 1108 participants included in the formal analysis, 618 were male and 490 were female. The age of participants ranged from 21 to 60 years, with a mean age of 40.63 years (*SD* = 9.54). Specifically, there were 481 young adults aged 40 and below, constituting 43.4% of the sample, while 627 middle-aged adults over the age of 40 comprised 56.6%. In terms of average daily AI product usage, 25.2% of employees reported using it for less than 10 min, 36.7% for 10–30 min, 18.2% for 30–60 min, and 19.9% for more than one hour.

### 2.2. Measurements

#### 2.2.1. AI Dependence

At Time 1, AI dependence was assessed using eight items adapted from the short version of the smartphone addiction scale (Kwon et al., 2013; Luk et al., 2018). These items target key features of problematic AI engagement, such as loss of control, withdrawal, and reduced efficiency. Sample items include “I rely too much on AI,” “I feel anxious and upset when I cannot use artificial intelligence,” and “I have tried to reduce the amount of time I spend on AI but failed.” Prior research has adopted the practice of adapting such items to measure AI dependence and has demonstrated good reliability and validity for the adapted scale (e.g., S. Huang et al., 2024). Participants rated their responses on a five-point scale, where a score of 1 represented the lowest level of dependence and a score of 5 indicated the highest level. Higher total scores reflect a more severe degree of AI dependence. In our study, the Cronbach’s alpha coefficient for the scale was 0.90, indicating strong internal consistency.

#### 2.2.2. Work Self-Efficacy

At Time 2, work self-efficacy was assessed using six items adapted from the General Self-Efficacy Scale (Schwarzer & Jerusalem, 1995), one of the most widely utilized measures of generalized self-efficacy, which has demonstrated strong psychometric properties across various cultural contexts (e.g., Das et al., 2024; Löve et al., 2012; Luszczynska et al., 2005). To better align the assessment with workplace dynamics, we tailored the scenarios in each item to reflect work-related contexts and instructed participants to evaluate these items based on their actual experiences within their professional environments. Sample items included, “I am always able to solve difficult problems when I put in the effort at work” and “I find it easy to adhere to my plans and achieve my objectives at work.” Participants rated the extent to which these statements accurately represented their work situations on a five-point response scale (1 = Does not fit; 5 = Fits perfectly), with higher total scores indicating greater levels of work self-efficacy. In our study, the Cronbach’s alpha coefficient for this scale was 0.93, indicating strong internal consistency.

#### 2.2.3. Work Engagement

At both Time 1 and Time 3, work engagement was evaluated using the Chinese version (Fong & Ng, 2012) of the nine-item Utrecht Work Engagement Scale (UWES-9, Schaufeli et al., 2006). The three dimensions of Vigor, Dedication, and Absorption were each measured with three items. Participants rated these items on a scale from 0 (never) to 6 (always). Higher scores indicate a greater level of work engagement. In our study, the Cronbach’s alpha coefficient for the scale was 0.95 at Time 1 and 0.94 at Time 2, indicating good internal consistency.

### 2.3. Analysis Strategies

Our research began with a Pearson correlation analysis to examine the relationships among AI dependence, work self-efficacy, and work engagement. Subsequently, we utilized the SPSS 23.0 macro PROCESS (Hayes, 2013) to analyze a mediation model that clarifies how T2 work self-efficacy mediates the longitudinal relationship between T1 AI dependence and T3 work engagement. Finally, we conducted a moderated mediation model analysis to assess whether gender moderates the direct and indirect associations between T1 AI dependence and T3 work engagement. During model testing, we incorporated potential confounding variables, including gender, age, AI use intensity, industry type (categorized as manufacturing, education, or internet companies) and job role category (categorized as ordinary employees, middle management, or senior management) as covariates to control for their possible impact on the results.

## 3. Results

### 3.1. Preliminary Analysis

The results of the correlation analysis are presented in Table 1. In both male and female groups, T1 AI dependence exhibited a negative association with T1 work engagement, T2 work self-efficacy, and T3 work engagement. In contrast, T2 work self-efficacy demonstrated a positive association with both T1 work engagement and T3 work engagement.

### 3.2. Testing for the Mediating Model

Table 2 displays the results of a mediation analysis examining the role of T2 work self-efficacy in the longitudinal relationship between T1 AI dependence and T3 work engagement among employees. The results reveal that T1 AI dependence significantly predicted T3 work engagement (β = −0.36, *p* < 0.001, 95% CI [−0.42, −0.31]) when mediators were not included, while controlling for gender, age, AI usage intensity, industry type, job role category, and T1 work engagement. Moreover, T1 AI dependence negatively predicted T2 work self-efficacy (β = −0.38, *p* < 0.001, 95% CI [−0.44, −0.32]). In the regression model that included the independent variable, mediating variable, and covariates, T2 work self-efficacy positively predicted T3 work engagement (β = 0.38, *p* < 0.001, 95% CI [0.33, 0.44]). Furthermore, the predictive association between T1 AI dependence and T3 work engagement remained significant (β = −0.22, *p* < 0.001, 95% CI [−0.27, −0.16]). Utilizing the bias-corrected percentile bootstrap method, the mediating effect of T2 work self-efficacy was estimated at −0.14 (Table 3), with a 95% confidence interval of [−0.18, −0.11], suggesting that it accounted for 39.82% of the total effect of AI dependence on work engagement.

### 3.3. Testing for the Moderated Mediation Model

Table 4 presents the results of a moderated mediation analysis, which examines whether gender moderates the direct path from T1 AI dependence to T3 work engagement and its indirect path through T2 work self-efficacy. The analysis indicates that the interaction between T1 AI dependence and gender did not significantly predict T2 work self-efficacy (β = 0.07, *p* = 0.34, 95% CI [−0.06, 0.19]). However, the interaction between T1 AI dependence and gender positively predicted T3 work engagement (β = 0.14, *p* < 0.05, 95% CI [0.04, 0.23]). These findings imply that gender did not moderate the indirect role of T2 work self-efficacy in the longitudinal relationship between AI dependence and work engagement; nonetheless, the direct longitudinal relationship demonstrated significant gender differences. The conditional effect analysis (Table 5) further revealed that the direct association between T1 AI dependence and T3 work engagement was stronger for males (β = −0.27, *p* < 0.001) compared to females (β = −0.14, *p* < 0.001). In contrast, the mediating effect of work self-efficacy exhibited no significant gender differences, with both males (β = −0.15, *p* < 0.001) and females (β = −0.13, *p* < 0.001) exhibiting relatively equivalent effects.

## 4. Discussion

In the era of artificial intelligence, AI dependence has emerged as a growing concern among researchers and the general public. While educational research has increasingly explored the effects of AI dependence on student populations, its implications in the workplace remain underexplored. The present study aims to extend previous research by analyzing longitudinal data collected at three time points over a one-year period, focusing on the lasting impact of AI dependence on employee work engagement. Additionally, we investigate work self-efficacy as a potential mediator and gender as a possible moderator within the longitudinal relationship between AI dependence and work engagement. After controlling for gender, age, AI usage intensity, industry type, job role category, and T1 work engagement, our analyses revealed three significant findings. First, T1 AI dependence exhibited a significant longitudinal effect on T3 work engagement. Second, T2 work self-efficacy significantly mediated the longitudinal relationship between T1 AI dependence and T3 work engagement. Third, while the mediational pathway was consistent across genders, we observed notable gender differences in the direct effects: male employees demonstrated a stronger longitudinal association between AI dependence and subsequent work engagement compared to their female counterparts. As one of the first systematic explorations of AI dependence in organizational contexts, our study makes important theoretical contributions by delineating the temporal dynamics and psychological mechanisms underlying the relationship between AI dependence and work engagement. Practically, these findings offer actionable insights for organizations striving to integrate AI while sustaining optimal employee engagement levels.

Consistent with Hypothesis 1, our study confirms that AI dependence has a significant and direct long-term predictive effect on employees’ work engagement, even after controlling for multiple potential confounding factors. The direct effect may stem from the undermining of intrinsic motivation caused by overreliance on AI. According to self-determination theory (Deci et al., 2017), autonomy, competence, and relatedness are three basic psychological needs whose satisfaction enhances intrinsic motivation and facilitates proactive engagement. However, when employees become dependent on AI and use it excessively in their work, the fulfillment of these basic psychological needs may be thwarted. For instance, overreliance on AI to complete tasks deprives employees of opportunities to achieve goals through their own efforts, thereby weakening the reinforcement of a sense of competence. Moreover, heavy dependence on AI may prevent employees from choosing and undertaking tasks that AI cannot handle, which may further impair the satisfaction of their need for autonomy. When both competence and autonomy are undermined, employees’ work enthusiasm and active engagement are likely to suffer. Therefore, the negative implications of excessive AI reliance on employees’ career development warrant serious attention.

Consistent with Hypothesis 2, our results confirm the mediating role of work self-efficacy between AI dependence and work engagement. Work self-efficacy reflects how fully an individual’s need for competence is met, wherein stronger self-efficacy indicates more complete satisfaction of that fundamental psychological need. The observed positive relationship between work self-efficacy and work engagement aligns with findings from numerous previous empirical studies (e.g., Chaudhary et al., 2012; Orgambídez et al., 2020; Song et al., 2018). However, the present study extends prior research by examining such a relationship within the specific context of AI dependence. Importantly, our analysis identifies work self-efficacy as a proximal mechanism through which AI dependence exerts its long-term effect on employee work engagement, thereby serving as a critical mediator in the overall process. The negative predictive effect of AI dependence on work self-efficacy may be explained through several possible mechanisms. First, AI dependence may contribute to technostress, particularly feelings of AI-related anxiety (Frenkenberg & Hochman, 2025). The broaden-and-build theory posits that positive emotions broaden and build enduring personal resources, whereas negative emotions impede their development (Fredrickson, 2001). As a potent negative emotion, anxiety is likely to disrupt the formation of key psychological resources, including self-efficacy. Empirical evidence also indicates that both general anxiety and subject-specific anxiety have a significant negative predictive effect on self-efficacy. (C. L. Lee & Huang, 2014; Palestro & Jameson, 2020; R. Zhang et al., 2023). Second, the negative influence of AI dependence on self-efficacy might originate from negative vicarious experiences. For example, when employees observe AI completing tasks more efficiently than themselves, they may develop negative observational learning beliefs, such as perceiving themselves as inferior to AI (Tufail et al., 2024), which can diminish their self-efficacy. Third, employees may attribute successful outcomes to AI rather than to their own efforts, while blaming themselves for failures. Such a negative attribution pattern may weaken their self-assessment of competence and indirectly reduce work engagement. It is crucial to acknowledge that although explanations such as technostress, feelings of incompetence, and threats to autonomy are theoretically plausible, they were not directly tested in our study and thus serve as speculative pathways requiring rigorous empirical validation.

Building upon the established direct effect of AI dependence and the mediating role of work self-efficacy, the present study further identified a significant moderating effect of gender. While the mediating pathway through work self-efficacy showed no significant gender differences—thus not supporting Hypothesis 4—the direct effect of AI dependence on work engagement was significantly stronger among male employees than among female employees, though statistically significant in both groups, supporting Hypothesis 3. The significant gender difference in the direct effect suggests that the negative association between AI dependence and work engagement was stronger among male employees. Self-determination theory posits that autonomy is a fundamental psychological need (Deci et al., 2017). To the extent that AI dependence compromises autonomy, it may reduce intrinsic motivation and engagement. This gender-specific pattern may be explained by the hypothesis that males might be more susceptible to such autonomy impairment. Within many traditional gender role frameworks, which emphasize assertiveness and control for men (Leaper & Ayres, 2007; Parham et al., 2015), reliance on AI for decision-making could be construed as a salient threat to personal agency, thus potentially accounting for their heightened vulnerability. Weakened autonomy is likely to lead to reduced intrinsic motivation (Guay et al., 2001; Meng & Ma, 2015) and lower work engagement (Malinowska et al., 2018). In addition, social identity theory and relevant empirical studies suggest that professional role identity is crucial for work engagement (Arshad et al., 2022; Q. Zhang et al., 2024; H. Zhang et al., 2022). A related hypothesis to explain the observed gender difference is that the erosion of role identity by AI dependence may follow gender-specific patterns. Specifically, it is plausible that men, who often more strongly associate professional identity with technological mastery and independent decision-making (Cech, 2015; Delaney et al., 2015), might perceive excessive AI reliance as inducing greater role ambiguity (e.g., uncertainty about their primary task) and, in turn, feelings of professional inadequacy. For them, such identity disruption could therefore be more detrimental to work engagement. In contrast, professional roles traditionally associated with women tend to emphasize relational coordination (Post, 2015), a domain less susceptible to full AI substitution. Consequently, even with considerable AI assistance, female employees may maintain a stronger perception of their own irreplaceability, experience less identity erosion, and consequently exhibit more stable levels of work engagement. It is important to emphasize that these gender-specific interpretations, while theoretically grounded, remain speculative. Future research is needed to directly test whether perceived threats to autonomy and professional identity may mediate the stronger negative association observed among men.

The present study has several limitations. First, although we employed a longitudinal design with data collected over a one-year period, the questionnaire-based nature of the research ultimately provides correlational rather than causal evidence. Therefore, we cannot firmly establish causal relationships among the variables. Future research could adopt intervention studies with quasi-experimental designs to examine whether reducing AI dependence leads to improvements in work self-efficacy and work engagement. Second, while the predictive effect of AI dependence on work engagement may operate through multiple complex mechanisms, we focused exclusively on the mediating role of work self-efficacy. Other potentially relevant mediating variables were not examined. Future studies should simultaneously test multiple indirect pathways and compare the relative strength of their mediating effects. Third, although advanced longitudinal methods (e.g., the random-intercept cross-lagged panel model) offer greater robustness for multi-wave data, our study design did not permit their application. To minimize participant burden and reduce attrition, the three core constructs (AI dependence, work self-efficacy, and work engagement) were not assessed at every wave. Consequently, our data structure does not meet the requirements for such modeling. Future research should incorporate these methodological considerations to enable stronger longitudinal analyses of AI-related phenomena. Fourth, our measure of AI dependence was adapted from a smartphone addiction scale. While it captures features of compulsive technology use, it may not fully operationalize the specific construct of reliance on AI for cognitive offloading or professional decision-making. Moreover, the lack of reported evidence for its dimensionality, measurement invariance across gender, test–retest reliability, and discriminant validity is a notable limitation. Future studies should prioritize using newly validated scales, such as the Generative AI Dependency Scale (Goh et al., 2025), or undertake similar scale development and validation efforts to ensure robust measurement of this nuanced form of AI-aided decision dependence.

Despite its limitations, our offers several important practical implications. First, given the stable and long-term negative impact of AI dependence on work engagement, organizations, as decision-makers and resource providers of AI technology, should develop comprehensive strategies at institutional, technological, and training levels to mitigate its detrimental effect on employee engagement. These organization-wide measures can help reduce excessive reliance on AI and preserve meaningful human involvement in work tasks. It is critical to qualify that our conclusions pertain specifically to excessive reliance on AI, not to its normative or productive use. When used appropriately, AI can serve as a valuable tool for facilitating complex tasks and enhancing engagement. Second, since work self-efficacy plays a critical mediating role in the relationship between AI dependence and work engagement, organizations can clearly delineate the scope of AI application (e.g., data filtering, repetitive operations) and emphasize core human responsibilities (e.g., strategy formulation, client communication, innovative design) through job descriptions and AI usage guidelines. The differentiation can help prevent AI from displacing tasks that require employees’ competence and agency. Third, while self-efficacy enhancement should be universally promoted, additional tailored interventions are needed to address higher AI dependence among male employees, because the negative direct effect of AI dependence on work engagement was stronger among them. For example, organizations could grant male employees greater autonomy over AI usage, such as allowing them to decide when and how to use AI tools and evaluate AI-generated outcomes. Such proactive involvement may help restore their sense of control and attenuate the decline in work engagement. Finally, as active agents in their career development, employees should consciously reflect on and adjust their relationship with AI. Shifting from passive dependency to active mastery can help maintain self-efficacy and sustain long-term work engagement.

## 5. Conclusions

We conducted a three-wave longitudinal study over one year to examine the long-term impact of AI dependence on employee work engagement, investigating both its direct and indirect pathways as well as potential gender differences in these effects. The results showed that AI dependence not only directly predicted work engagement one year later but also indirectly predicted it through work self-efficacy measured at the six-month interval. Furthermore, while the mediating effect of work self-efficacy showed no significant gender differences, the direct effect of AI dependence on work engagement was significantly stronger among male employees than among female employees. As one of the first studies to systematically analyze the long-term effects, underlying mechanisms, and boundary conditions of AI dependence on employee work engagement, our findings offer critical warnings regarding the prevention of excessive AI reliance in organizational settings and provide practical insights for preserving and enhancing employees’ work self-efficacy and engagement.

## Figures and Tables

**Table 1:** Descriptive statistics and correlations between variables.

Variables	*M* (*SD*) for Males	*M* (*SD*) for Females	1	2	3	4
1. T1 AI dependence	19.77 (5.43)	19.97 (4.81)	—	−0.14 ***	−0.33 ***	−0.29 ***
2. T1 Work engagement	34.30 (11.03)	35.06 (10.54)	−0.31 ***	—	0.14 ***	0.33 ***
3. T2 Work self-efficacy	18.86 (3.77)	18.85 (3.65)	−0.47 ***	0.36 ***	—	0.50 ***
4. T3 Work engagement	36.35 (11.39)	36.57 (10.15)	−0.53 ***	0.52 ***	0.62 ***	—

Note. *N* = 1108. *** *p* < 0.01.

**Table 2:** Mediation analysis of work self-efficacy.

Regression Equation		Significance of Regression Coefficients				Bootstrap	
Dependent Variables	Independent Variables	β	*SE*	*t*	*p*	LLCI	ULCI
T3 Work engagement	Gender	0.02	0.05	0.46	0.64	−0.08	0.13
	Age	0.17 ***	0.03	5.68	<0.001	0.11	0.23
	AI use intensity	0.06 *	0.03	2.20	<0.05	0.01	0.11
	Industry category	−0.08 ***	0.02	−4.48	<0.001	−0.12	−0.05
	Job role category	0.01	0.07	0.17	0.87	−0.13	0.15
	T1 Work engagement	0.33 ***	0.03	12.50	<0.001	0.28	0.39
	T1 AI dependence	−0.36 ***	0.03	−13.75	<0.001	−0.42	−0.31
T2 Work self-efficacy	Gender	0.07	0.06	1.15	0.25	−0.05	0.18
	Age	0.16 ***	0.03	4.68	<0.001	0.09	0.23
	AI use intensity	0.06 *	0.03	2.07	<0.05	0.01	0.11
	Industry category	−0.04 *	0.02	−2.06	<0.05	−0.08	−0.01
	Job role category	−0.16 *	0.08	−1.98	<0.05	−0.33	−0.01
	T1 Work engagement	0.17 ***	0.03	5.31	<0.001	0.10	0.23
	T1 AI dependence	−0.38 ***	0.03	−11.99	<0.001	−0.44	−0.32
T3 Work engagement	Gender	−0.01	0.05	−0.03	0.98	−0.10	0.09
	Age	0.11 ***	0.03	3.85	<0.001	0.06	0.17
	AI use intensity	0.03	0.02	1.47	0.14	−0.01	0.08
	Industry category	−0.07	0.02	−4.00	<0.001	−0.10	−0.03
	Job role category	0.07	0.07	1.15	0.25	−0.05	0.20
	T1 Work engagement	0.27 ***	0.03	10.27	<0.001	0.22	0.32
	T1 AI dependence	−0.22 ***	0.03	−7.96	<0.001	−0.27	−0.16
	T2 Work self-efficacy	0.38 ***	0.03	13.99	<0.001	0.33	0.44

Note. *N* = 1108. Bootstrap sample size = 5000. LL = low limit, CI = confidence interval, UL = upper limit. * *p* < 0.05. *** *p* < 0.001.

**Table 3:** Total, direct, and indirect effects.

Effects	β	*SE*	Bootstrap	
			LLCI	ULCI
Total effect	−0.36 ***	0.03	−0.41	−0.31
Direct effect	−0.22 ***	0.03	−0.27	−0.16
Indirect path: AI dependence→work self-efficacy→work engagement	−0.14 ***	0.02	−0.18	−0.11

Note. *N* = 1108. Bootstrap sample size = 5000. LL = low limit, CI = confidence interval, UL = upper limit. *** *p* < 0.001.

**Table 4:** Moderated mediation analysis of work self-efficacy and gender.

Regression Equation		Significance of Regression Coefficients				Bootstrap	
Dependent Variables	Independent Variables	β	*SE*	*t*	*p*	LLCI	ULCI
T2 Work self-efficacy	Gender	0.07	0.06	1.17	0.24	−0.05	0.19
	Age	0.16 ***	0.03	4.69	<0.001	0.09	0.23
	AI use intensity	0.06 *	0.03	2.04	<0.05	0.01	0.12
	Industry category	−0.04 *	0.02	−2.06	<0.05	−0.08	−0.01
	Job role category	−0.17 *	0.08	−2.04	<0.05	−0.33	−0.01
	T1 Work engagement	0.16 ***	0.03	5.23	<0.001	0.10	0.22
	T1 AI dependence	−0.37 ***	0.03	−11.92	<0.001	−0.44	−0.31
	T1 AI dependence × Gender	0.07	0.06	1.04	0.30	−0.06	0.19
T3 Work engagement	Gender	0.01	0.05	0.04	0.97	−0.09	0.10
	Age	0.11 ***	0.03	3.88	<0.001	0.06	0.17
	AI use intensity	0.03	0.03	1.45	0.15	−0.01	0.08
	Industry category	−0.06 ***	0.02	−3.99	<0.001	−0.10	−0.03
	Job role category	0.06	0.07	0.97	0.33	−0.06	0.19
	T1 Work engagement	0.27 ***	0.03	10.09	<0.001	0.21	0.32
	T1 AI dependence	−0.21 ***	0.03	−7.64	<0.001	−0.27	−0.16
	T2 Work self-efficacy	0.38 ***	0.03	13.90	<0.001	0.33	0.44
	T1 AI dependence × Gender	0.14 **	0.05	2.75	<0.01	0.04	0.23

Note. *N* = 1108. Bootstrap sample size = 5000. LL = low limit, CI = confidence interval, UL = upper limit. * *p* < 0.05. ** *p* < 0.01. *** *p* < 0.001.

**Table 5:** Conditional effect analysis.

Conditional direct effect analysis at different genders	β	SE	Boot LLCI	Boot ULCI
Males	−0.27 ***	0.03	−0.33	−0.21
Female	−0.14 **	0.04	−0.23	−0.05
**Conditional indirect effect analysis at different genders**	**β**	**SE**	**Boot LLCI**	**Boot ULCI**
Males	−0.15 ***	0.02	−0.19	−0.11
Females	−0.13 ***	0.02	−0.17	−0.09

Note. *N* = 1108. Bootstrap sample size = 5000. LL = low limit, CI = confidence interval, UL = upper limit. ** *p* < 0.010. *** *p* < 0.001.

## Data Availability

The data are not publicly available due to privacy and ethical restrictions. The data that support the findings of this study are available on reasonable request from the corresponding author following the completion of a privacy and fair use agreement.

## References

[B1-behavsci-16-00044] Arora M. (2025). A study on association between AI and critical thinking, impulsivity, dependence among young adults. International Journal of Interdisciplinary Approaches in Psychology.

[B2-behavsci-16-00044] Arshad M., Qasim N., Farooq O., Rice J. (2022). Empowering leadership and employees’ work engagement: A social identity theory perspective. Management Decision.

[B3-behavsci-16-00044] Bakker A. B., Demerouti E. (2008). Towards a model of work engagement. Career Development International.

[B4-behavsci-16-00044] Balducci C., Fraccaroli F., Schaufeli W. B. (2010). Psychometric properties of the Italian version of the Utrecht Work Engagement Scale (UWES-9). European Journal of Psychological Assessment.

[B5-behavsci-16-00044] Bandura A. (2001). Social cognitive theory: An agentic perspective. Annual Review of Psychology.

[B6-behavsci-16-00044] Bandura A., Urdan T., Pajares F. (2006). Guide for constructing self-efficacy scales. Self-efficacy beliefs of adolescents.

[B7-behavsci-16-00044] Banihani M., Lewis P., Syed J. (2013). Is work engagement gendered?. Gender in Management: An International Journal.

[B8-behavsci-16-00044] Boukari Z. I., Elseesy N. A. M., Felemban O., Alharazi R. (2025). Between clicks and care: Investigating social media addiction and work engagement among nurses in Saudi Arabia. Nursing Reports.

[B9-behavsci-16-00044] Caesens G., Stinglhamber F. (2014). The relationship between perceived organizational support and work engagement: The role of self-efficacy and its outcomes. European Review of Applied Psychology.

[B10-behavsci-16-00044] Caliskan F., Idug Y., Uvet H., Gligor N., Kayaalp A. (2024). Social comparison theory: A review and future directions. Psychology & Marketing.

[B11-behavsci-16-00044] Cech E. (2015). Engineers and engineeresses? Self-conceptions and the development of gendered professional identities. Sociological Perspectives.

[B12-behavsci-16-00044] Chaudhary R., Rangnekar S., Barua M. K. (2012). Relationships between occupational self efficacy, human resource development climate, and work engagement. Team Performance Management: An International Journal.

[B13-behavsci-16-00044] Das S. K., Philip M., Sudhir P. M., VS B. (2024). Psychometric evaluation of Schwarzer & Jerusalem’s General Self-Efficacy Scale among Indian adolescents: A factor analysis and multidimensional item response theory approach. Measurement Instruments for the Social Sciences.

[B14-behavsci-16-00044] Deci E. L., Olafsen A. H., Ryan R. M. (2017). Self-determination theory in work organizations: The state of a science. Annual Review of Organizational Psychology and Organizational Behavior.

[B15-behavsci-16-00044] Delaney R., Strough J., Parker A. M., de Bruin W. B. (2015). Variations in decision-making profiles by age and gender: A cluster-analytic approach. Personality and Individual Differences.

[B16-behavsci-16-00044] Festinger L. (1954). A theory of social comparison processes. Human Relations.

[B17-behavsci-16-00044] Fong T. C. T., Ng S. M. (2012). Measuring engagement at work: Validation of the Chinese version of the Utrecht Work Engagement Scale. International Journal of Behavioral Medicine.

[B18-behavsci-16-00044] Fredrickson B. L. (2001). The role of positive emotions in positive psychology: The broaden-and-build theory of positive emotions. American Psychologist.

[B19-behavsci-16-00044] Frenkenberg A., Hochman G. (2025). It’s scary to use it, it’s scary to refuse it: The psychological dimensions of AI adoption—Anxiety, motives, and dependency. Systems.

[B20-behavsci-16-00044] George A. S., Baskar T., Srikaanth P. B. (2024). The erosion of cognitive skills in the technological age: How reliance on technology impacts critical thinking, problem-solving, and creativity. Partners Universal Innovative Research Publication.

[B21-behavsci-16-00044] Goh A. Y., Hartanto A., Majeed N. M. (2025). Generative artificial intelligence dependency: Scale development, validation, and its motivational, behavioral, and psychological correlates. Computers in Human Behavior Reports.

[B22-behavsci-16-00044] Guay F., Boggiano A. K., Vallerand R. J. (2001). Autonomy support, intrinsic motivation, and perceived competence: Conceptual and empirical linkages. Personality and Social Psychology Bulletin.

[B23-behavsci-16-00044] Hayes A. F. (2013). Introduction to mediation, moderation, and conditional process analysis: A regression-based approach.

[B24-behavsci-16-00044] Hobfoll S. E., Halbesleben J., Neveu J. P., Westman M. (2018). Conservation of resources in the organizational context: The reality of resources and their consequences. Annual Review of Organizational Psychology and Organizational Behavior.

[B25-behavsci-16-00044] Hu B., Mao Y., Kim K. J. (2023). How social anxiety leads to problematic use of conversational AI: The roles of loneliness, rumination, and mind perception. Computers in Human Behavior.

[B26-behavsci-16-00044] Huang J., Huang M. T., Wang F. (2024). Social media addiction and employees’ innovative behavior: A moderated mediation model of work engagement and mindfulness. Current Psychology.

[B27-behavsci-16-00044] Huang S., Lai X., Ke L., Li Y., Wang H., Zhao X., Dai X., Wang Y. (2024). AI technology panic—Is AI dependence bad for mental health? A cross-lagged panel model and the mediating roles of motivations for AI use among adolescents. Psychology Research and Behavior Management.

[B28-behavsci-16-00044] Ibrahim M., Yusra Y., Shah N. U. (2022). Impact of social media addiction on work engagement and job performance. Polish Journal of Management Studies.

[B29-behavsci-16-00044] Khan A. N., Moin M. F., Khan N. A., Zhang C. (2022). A multistudy analysis of abusive supervision and social network service addiction on employee’s job engagement and innovative work behaviour. Creativity and Innovation Management.

[B30-behavsci-16-00044] Kwon M., Kim D. J., Cho H., Yang S. (2013). The smartphone addiction scale: Development and validation of a short version for adolescents. PLoS ONE.

[B31-behavsci-16-00044] Laestadius L., Bishop A., Gonzalez M., Illenčík D., Campos-Castillo C. (2024). Too human and not human enough: A grounded theory analysis of mental health harms from emotional dependence on the social chatbot Replika. New Media & Society.

[B32-behavsci-16-00044] Leaper C., Ayres M. M. (2007). A meta-analytic review of gender variations in adults’ language use: Talkativeness, affiliative speech, and assertive speech. Personality and Social Psychology Review.

[B33-behavsci-16-00044] Lee C. L., Huang M. K. (2014). The influence of computer literacy and computer anxiety on computer self-efficacy: The moderating effect of gender. Cyberpsychology, Behavior, and Social Networking.

[B34-behavsci-16-00044] Lee H. P., Sarkar A., Tankelevitch L., Drosos I., Rintel S., Banks R., Wilson N. (2025). The impact of generative AI on critical thinking: Self-reported reductions in cognitive effort and confidence effects from a survey of knowledge workers. Proceedings of the 2025 CHI conference on Human Factors in Computing Systems.

[B35-behavsci-16-00044] Lin H., Chen Q. (2024). Artificial intelligence integrated educational applications and college students’ creativity and academic emotions: Students and teachers’ perceptions and attitudes. BMC Psychology.

[B36-behavsci-16-00044] Lloyd J., Bond F. W., Flaxman P. E. (2017). Work-related self-efficacy as a moderator of the impact of a worksite stress management training intervention: Intrinsic work motivation as a higher order condition of effect. Journal of Occupational Health Psychology.

[B37-behavsci-16-00044] Locke E. A., Latham G. P. (2006). New directions in goal-setting theory. Current Directions in Psychological Science.

[B38-behavsci-16-00044] Locke E. A., Latham G. P. (2019). The development of goal setting theory: A half century retrospective. Motivation Science.

[B39-behavsci-16-00044] Löve J., Moore C. D., Hensing G. (2012). Validation of the Swedish translation of the general self-efficacy scale. Quality of Life Research.

[B40-behavsci-16-00044] Lu L., Wang L. (2023). When mothers are more negative while fathers are less positive: Offspring’s temporary feelings of depression affect parental work engagement via the asymmetric effects of emotions transmission. PsyCh Journal.

[B41-behavsci-16-00044] Luk T. T., Wang M. P., Shen C., Wan A., Chau P. H., Oliffe J., Viswanath K., Chan S. S. C., Lam T. H. (2018). Short version of the Smartphone Addiction Scale in Chinese adults: Psychometric properties, sociodemographic, and health behavioral correlates. Journal of Behavioral Addictions.

[B42-behavsci-16-00044] Luszczynska A., Scholz U., Schwarzer R. (2005). The general self-efficacy scale: Multicultural validation studies. The Journal of Psychology.

[B43-behavsci-16-00044] Malinowska D., Tokarz A., Wardzichowska A. (2018). Job autonomy in relation to work engagement and workaholism: Mediation of autonomous and controlled work motivation. International Journal of Occupational Medicine and Environmental Health.

[B44-behavsci-16-00044] Meng L., Ma Q. (2015). Live as we choose: The role of autonomy support in facilitating intrinsic motivation. International Journal of Psychophysiology.

[B45-behavsci-16-00044] Metin Camgoz S., Tayfur Ekmekci O., Bayhan Karapinar P., Kumbul Guler B. (2016). Job insecurity and turnover intentions: Gender differences and the mediating role of work engagement. Sex Roles.

[B46-behavsci-16-00044] Mills M. J., Culbertson S. S., Fullagar C. J. (2012). Conceptualizing and measuring engagement: An analysis of the Utrecht Work Engagement Scale. Journal of Happiness Studies.

[B47-behavsci-16-00044] Morales-García W. C., Sairitupa-Sanchez L. Z., Morales-García S. B., Morales-García M. (2024). Development and validation of a scale for dependence on artificial intelligence in university students. Frontiers in Education.

[B48-behavsci-16-00044] Mukhtar M., Firdos S. S., Zaka I., Naeem S. (2025). Impact of AI dependence on procrastination among university students. Research Journal of Psychology.

[B49-behavsci-16-00044] Naseer A., Ahmad N. R., Chishti M. A. (2025). Psychological impacts of AI dependence: Assessing the cognitive and emotional costs of intelligent systems in daily life. Review of Applied Management and Social Sciences.

[B50-behavsci-16-00044] Orgambídez A., Borrego Y., Vázquez-Aguado O. (2020). Linking self-efficacy to quality of working life: The role of work engagement. Western Journal of Nursing Research.

[B51-behavsci-16-00044] Palestro J. J., Jameson M. M. (2020). Math self-efficacy, not emotional self-efficacy, mediates the math anxiety-performance relationship in undergraduate students. Cognition, Brain, Behavior.

[B52-behavsci-16-00044] Parham J. B., Lewis C. C., Fretwell C. E., Irwin J. G., Schrimsher M. R. (2015). Influences on assertiveness: Gender, national culture, and ethnicity. Journal of Management Development.

[B53-behavsci-16-00044] Pei S., Wang S., Jiang R., Guo J., Ni J. (2024). How work stress influence turnover intention among Chinese local undergraduate university teachers: The mediating effect of job burnout and the moderating effect of self-efficacy. Frontiers in Public Health.

[B54-behavsci-16-00044] Post C. (2015). When is female leadership an advantage? Coordination requirements, team cohesion, and team interaction norms. Journal of Organizational Behavior.

[B55-behavsci-16-00044] Raelin J. A., Bailey M., Hamann J., Pendleton L., Raelin J., Reisberg R., Whitman D. (2011). The effect of cooperative education on change in self-efficacy among undergraduate students: Introducing work self-efficacy. Journal of Cooperative Education and Internships.

[B56-behavsci-16-00044] Rafiei S., Souri S., Nejatifar Z., Amerzadeh M. (2024). The moderating role of self-efficacy in the relationship between occupational stress and mental health issues among nurses. Scientific Reports.

[B57-behavsci-16-00044] Rožman M., Sternad Zabukovšek S., Bobek S., Tominc P. (2021). Gender differences in work satisfaction, work engagement and work efficiency of employees during the COVID-19 pandemic: The case in Slovenia. Sustainability.

[B58-behavsci-16-00044] Schaufeli W. B., Bakker A. B., Bakker A. B., Leiter M. P. (2010). Defining and measuring work engagement: Bringing clarity to the concept. Work engagement: A handbook of essential theory and research.

[B59-behavsci-16-00044] Schaufeli W. B., Bakker A. B., Salanova M. (2006). The measurement of work engagement with a short questionnaire: A cross-national study. Educational and Psychological Measurement.

[B60-behavsci-16-00044] Schwarzer R., Jerusalem M., Weinman J., Wright S., Johnston M. (1995). Generalized Self-Efficacy scale. Measures in health psychology: A user’s portfolio. Causal and control beliefs.

[B61-behavsci-16-00044] Shimazu A., Schaufeli W. B., Kosugi S., Suzuki A., Nashiwa H., Kato A., Sakamoto M., Irimajiri H., Amano S., Hirohata K., Kitaoka-Higashiguchi K. (2008). Work engagement in Japan: Validation of the Japanese version of the Utrecht Work Engagement Scale. Applied Psychology.

[B62-behavsci-16-00044] Song J. H., Chai D. S., Kim J., Bae S. H. (2018). Job performance in the learning organization: The mediating impacts of self-efficacy and work engagement. Performance Improvement Quarterly.

[B63-behavsci-16-00044] Steyn R., Grobler S. (2016). Sex differences and work engagement: A study across 27 South African companies. Journal of Contemporary Management.

[B64-behavsci-16-00044] Tian G., Pu L., Ren H. (2021). Gender differences in the effect of workplace loneliness on organizational citizenship behaviors mediated by work engagement. Psychology Research and Behavior Management.

[B65-behavsci-16-00044] Tian G., Wang J., Zhang Z., Wen Y. (2019). Self-efficacy and work performance: The role of work engagement. Social Behavior and Personality: An International Journal.

[B66-behavsci-16-00044] Tufail R., Shahwani A. M., Khan W., Badar Y. (2024). Examining the impact of AI-generated content on self-esteem and body image through social comparison. Bulletin of Business and Economics.

[B67-behavsci-16-00044] Yankouskaya A., Liebherr M., Ali R. (2025). Can ChatGPT Be addictive? A call to examine the shift from support to dependence in AI conversational large language models. Human-Centric Intelligent Systems.

[B68-behavsci-16-00044] Zhai C., Wibowo S., Li L. D. (2024). The effects of over-reliance on AI dialogue systems on students’ cognitive abilities: A systematic review. Smart Learning Environments.

[B69-behavsci-16-00044] Zhang D., Wijaya T. T., Wang Y., Su M., Li X., Damayanti N. W. (2025). Exploring the relationship between AI literacy, AI trust, AI dependency, and 21st century skills in preservice mathematics teachers. Scientific Reports.

[B70-behavsci-16-00044] Zhang H., Li H., Tian W., Liu W., Yang Y. (2022). The influence of professional identity on work engagement among nurses working in nursing homes in China. Journal of Nursing Management.

[B71-behavsci-16-00044] Zhang Q., Li W., Gao J., Sun B., Lin S. (2024). Teachers’ professional identity and job burnout: The mediating roles of work engagement and psychological capital. Psychology in the Schools.

[B72-behavsci-16-00044] Zhang R., Ma Q., Guan D. (2023). The impact of financial scarcity on green consumption: Sequential mediating effects of anxiety and self-efficacy. Psychology & Marketing.

[B73-behavsci-16-00044] Zhang X., Li Z., Zhang M., Yin M., Yang Z., Gao D., Li H. (2025). Exploring artificial intelligence (AI) Chatbot usage behaviors and their association with mental health outcomes in Chinese university students. Journal of Affective Disorders.

[B74-behavsci-16-00044] Zhao H., Ma Y., Chen Y. (2025). Facing or avoiding? How dependence on artificial intelligence influences hotel employees’ job crafting. International Journal of Contemporary Hospitality Management.

